# Identifying emotional variations in Spanish oncology patients during treatment decision consultations: a qualitative study

**DOI:** 10.1136/bmjopen-2025-099918

**Published:** 2026-02-02

**Authors:** Yeray Sañudo, Laura Aguado-González, Alba Medina Castillo, Jorge Sierra-Pérez

**Affiliations:** 1 Aragon Institute for Engineering Research (i3a), Department of Engineering Design and Manufacturing, University of Zaragoza, Zaragoza, Aragon, Spain; 2 Water and Environmental Health-IUCA Research Group, University of Zaragoza, Zaragoza, Spain; 3 Health Research Institute Aragón, Zaragoza, Aragon, Spain

**Keywords:** ONCOLOGY, Behavior, Health Services, Adult oncology, Patient-Centered Care, Patients

## Abstract

**Abstract:**

**Objectives:**

This qualitative study aims to identify which themes cause the most emotional variation in patients’ decisions about cancer treatment. Patients’ emotional variations are analysed to detect negative and positive variations to certain themes during the consultations. This analysis helps to detect what themes or elements affect patients’ experiences.

**Design and settings:**

A total of 31 clinical consultations from cancer care pathways were recorded, transcribed and analysed. Patients were followed up until they decided and started treatment for their cancer. An inductive content analysis is followed to interpret patients’ emotional variations. All patients were from Hospital Universitario Miguel Servet of Zaragoza, Spain.

**Participants:**

Twelve patients participated in the study, consisting of eight with breast cancer and four with melanoma. Of these patients, nine were female and three were male. Eligible participants were required to be new to the care pathways or to have used them more than 10 years ago. Participants were also required to be aged 18 or older, be fluent in Spanish and be available to participate for 3 months. Participants were excluded if the authors or oncologists noticed poor health conditions that could be exacerbated by the stress and additional effort involved in participating in the study.

**Results:**

Patients’ emotional variations identify five main themes: clinical history, cancer diagnosis, discussion of possible treatment choice, side effects (possible side effects of the selected treatment) and next steps (require complementary medical tests and more consultations to decide the treatment). Most of the emotional variations detected were negative and were mostly grouped into the categories of treatment, side effects and next steps. The most pronounced negative variations were related to delays in starting treatment and the possibility of hair loss.

**Conclusions:**

The themes and emotional variations identified in this study can help to understand patients’ experiences during their initial oncology consultations. These results represent a significant step towards enhancing communication and the patient experience within oncology practices. Oncologists could use these data and procedures to identify where patients experience negative emotions and respond appropriately.

**STRENGTHS AND LIMITATIONS OF THIS STUDY**

- It is a single-centre study, which allows for greater analysis of the context but makes it difficult to transfer to other settings.
- This study analyses the emotional variations of patients during their first oncology consultations, providing further insights into the patients’ experience.
- Given that researchers have assessed emotional reactions, there may be interpretation biases.
- Yardley’s criteria and SRQR (Standards for Reporting Qualitative Research) were followed to ensure the qualitative research quality.

## Introduction

 Cancer is the main health issue worldwide and the second leading cause of death.^1^ More than 19 million people were diagnosed with cancer in 2022,^2^ and the risk of developing cancer between 0 and 74 years is 20.2%.^3^ Fortunately, the mortality rate is declining due to treatment development.^1 4^ However, their side effects negatively impact patients’ quality of life.^5^ Patients may experience physical and psychological distress due to their curative treatment.^6^ As the public is widely aware of these side effects, they can generate fear and anxiety, particularly during initial oncological consultations.^7^ Patients must cope with the uncertainty of the diagnosis of a potentially fatal illness and treatment side effects,^8^ which affect their emotional state.^9^ Patients feel vulnerable during these consultations, and oncologists should handle these conversations with care.^7^

Patients’ emotions can affect their perception of the quality of service and their satisfaction, both of which are crucial to improving the patient experience.^10^ Measuring emotions can provide valuable insight into how to improve the patient experience. Nevertheless, emotions are a widely known concept that is the subject of some debate in the scientific literature. They can be defined in various ways; a recent definition describes them as positive or negative responses to external stimuli that follow repeating patterns occurring in a specific manner.^11 12^ Different emotion representation models exist, which can be categorised as discrete emotions or dimensional spaces.^13 14^ Dimensional models allow researchers to analyse emotional variations according to various dimensional scales (valence, arousal and dominance). This model is employed in various fields since they are easy to adapt to any context.^15^ Dimensions provide continuous scales for representing emotions, offering a deeper understanding and analysis.^16^

There is limited understanding of patients’ emotional variations during their first oncology consultations.^17^ This qualitative study aims to explore patients’ emotional variations throughout their first oncological consultations to inform which topics discussed create negative or positive emotional variations. The study provides insights to improve patient-centre care (PCC) and discussions between patients and clinicians.^18 19^

Psychological readiness is crucial for PCC and Shared Decision-Making (SDM).^20^,^22^ The vast majority of patients want to be involved in decisions relating to their treatments.^23^ As Keij *et al*^24^ stated, patients need to be psychologically ready to decide, and that having all the information is not enough. Negative emotions during initial consultations can lead to serious psychological consequences (eg, depression, anxiety, stress, etc) which can affect patients’ ability to cope with cancer and subsequently influence their decision-making processes.^25 26^

To our knowledge, the majority of qualitative studies analysing the patient experience have used interviews or focus groups.^717 27^,^29^ While these methods allow substantial qualitative data to be collected, the information obtained is inherently limited by patients’ memory (recall bias), the social dynamics of focus groups and moderator bias.^30 31^ Consequently, analysing the actual discourse during the consultations could provide further insights into how patients respond and cope with the discussed themes (eg, treatment, side effects, diagnosis). Numerous methods based on ethnography and service design techniques can be used to analyse this, such as shadowing and transcription analysis.^32^ These methods allow a comprehensive understanding of complex care pathways, providing multiple perspectives of the experience.^33^ However, they may lead to gaps in data collection, as different aspects can occur simultaneously.^34^ Transcription analysis is becoming increasingly important in qualitative analysis to conduct an analysis of discussions between patients and physicians. While it is a robust method, some limitations have to be considered (ie, transcription and interpretability bias).^35^

Oncological consultations should provide a safe space for patients to express their emotions and to receive responses to their concerns.^28^ Patients may experience relief when discussing emotional concerns with oncologists.^36^ Numerous studies have shown that empathic responses to patients’ negative emotions can lead to positive coping mechanisms.^37^ However, other studies have shown that doctors often avoid such responses for fear of wasting consultation time or becoming emotionally involved.^38 39^ Kennifer *et al*^40^ identified a significant gap in oncologists’ responses to patients expressing negative emotions in advanced cancer. They found that even when the patients expressed negative emotions, clinicians seemed to have difficulty identifying and responding emphatically. Aligned with other studies,^41^ oncologists only respond when patients express intense negative emotions. Oncologists should receive training to identify negative emotions. Understanding the causes of patients’ emotional variations can help train doctors to manage these variations more effectively.^42^

Furber *et al*^17^ analysed how patients experience their first oncology consultation, focusing on the information provided by the oncologists. They concluded that patients’ emotional variations directly affect their understanding of information about their illness. The authors found patients seemed to respond emotionally when they did not understand the information provided.

Compared with the previous studies, this new qualitative study yields insight into patients’ emotional reactions to the various themes discussed during consultations. As this study involved analysing audio transcriptions, the findings provide information on the exact moment the emotional reaction occurs. Furthermore, they will be able to provide more details about aspects that patients may not remember in a possible subsequent interview.

### Aim of the study

This novel qualitative study aims to understand how patients experience oncological treatment decision consultations. Understanding their emotional variations will help to know what themes generate emotional changes that affect their experience with the process and its perceived quality.

#### Objectives of the study

- To explore the patient’s decision-making process through qualitative analysis of their experiences and positive and negative emotional variations. Using emotional measures extracted from oncology consultations through to treatment decisions.
- To explore the barriers and facilitators that influence their experience through their emotional variations. This understanding will highlight which themes and moments oncologists should prioritise when communicating with patients, particularly during the process of assimilating information about their illness.

## Methods

### Study design

For this qualitative study, we employed an inductive content analysis, which is widely used in the field of healthcare. It is used not only to identify themes in unstructured data, but also to explore and interpret their meanings in depth.^43^ To do so, the authors adopted an interpretative approach, concerned with context variables and aimed to enrich insights. This approach enables a deeper exploration of individual experiences by considering different factors such as behavioural aspects based on participants’ experiences.^44^

For transparency, the study team comprised three PhD students (two design engineering PhD (YS, LA-G) and one Nurse PhD student (AMC)), and an industrial design Associate Professor (JS-P). All of them have expertise in health and patient experiences and have been trained in multidimensional emotional analysis. Additionally, this study is conducted as part of a European Project that focuses on the analysis and redesign of melanoma and breast cancer care pathways.

### Participants' recruitment

Recruitment was performed via the oncology department at the ‘Hospital Universitario Miguel Servet’ in Zaragoza, the reference hospital for Aragón, Spain. All the oncologists involved in the breast and melanoma cancer care pathway were notified and gave their consent to follow any patient who met the requirements. Their treating oncologists and AMC acted as gatekeepers with the patients and recruitment.

Eligible participants were required to be either new patients with cancer or patients who had not used the oncology service in the previous 10 years. This was so that they would not be familiar with the current process and would have no prior expectations. Participants were also required to be 18 years or older, fluent in Spanish and available to participate for 3 months. Participants were excluded if the researchers or oncologists noticed poor health conditions that could be aggravated by the stress and additional effort involved in participating in the study.

Patient recruitment was carried out in two steps. First, their responsible oncologist contacted them by phone prior to the first consultation, explained the details of the study and asked them to consider participation. Second, interested patients were asked to arrive 30 min before their oncology consultation. Patients were interviewed by AMC to receive a more detailed explanation and sign the consent form. It was made clear that they could leave the study at any time, without having to give any kind of explanation. Patients have time to discuss their participation with their relatives. All patients were aware of their cancer diagnosis before they visited the oncology department, their consultation aimed to complete the diagnostic and/or initiate treatment.

The sample size was determined during the study until data saturation was reached. Initially, it was anticipated that 20 participants would be required; however, data saturation was achieved with 10 patients. To ensure that no new information emerged, an additional two participants were followed up. The final number of samples is justified by the Information Power model, as each participant provided significant insights, which consequently reduced the total number of participants needed.^45^

### Data collection

Consultations between patients and oncologists were recorded from the first oncology consultation until a treatment decision was made for each patient. The number of consultations may vary between patients. Two to three consultations may be necessary before reaching a decision. All oncologists followed the same standardised hospital protocol, addressing the same topics and informing key aspects. The time for decision-making and treatment initiation is approximately 2.5 weeks per patient.

The recordings were made using a voice recorder from May to November 2024. Consultations were shadowed by one of the authors who stayed in the room and took notes on the patients’ corporal reactions (used in a sister study).^34^ This researcher was stationed in a corner of the office. They tried to be as inconspicuous as possible, interfering with the discussion between the patient and doctor. They left the office when physical examinations were performed. Consequently, every precaution was taken to make the participants as comfortable as possible.^46^ The same procedure was followed for the various consultations needed to make the treatment decision. Wherever possible, the same researcher remained with the patient to avoid the discomfort of having a different researcher for each consultation.

### Ethical considerations

Written informed consent was obtained from all participants prior to their inclusion. Additionally, the ethical considerations to be included in the study protocol were reviewed and analysed.^46^ Verbal consent was obtained at each observational session to confirm their continued agreement to participate, ensuring an ongoing informed consent process.

The data were treated confidentially and anonymised in compliance with Spain’s Organic Law 3/2018 on the protection of personal data and the General Data Protection Regulation (GDPR) (Regulation (EU) 2016/679). All researchers involved in the study signed a data confidentiality agreement.

### Data processing and analysis

This qualitative study methodology was underpinned by a contextual and emotional analysis of audio-recorded oncological consultations. The authors collaborated to create an iterative codebook to detect and analyse variations in patients’ emotional responses and the context. Rules and examples have pertained to three analytical goals: (1) identifying negative, neutral or positive emotional verbal variations; (2) measuring the severity of these variations; and (3) identifying the theme and the cause of the variation (table 1). Once the codebook had been created, authors YS and LA-G analysed each consultation transcription. First, they identified emotional phrases expressed by the patients (ie, ‘This situation is new and tough for us.’). Second, emotional variations were classified on a valence scale (table 1). This method of emotional analysis was chosen because it was thought to facilitate agreement among the researchers. Third, the context of each phrase was classified according to its theme (ie, ‘I will lose my hair’ theme=treatment side effect). Finally, the two coders compared their analyses, retaining only the sentences they both marked and reaching consensus on any discrepancies in the code, if necessary. Kappa statistics were 0.71 for emotional expressions, which indicates substantial agreement.

**Table 1:** Codebook and examples

Categories	Examples
**Emotional variations**	
1. Very negative	‘I can’t even look at myself in the mirror.’
2. Negative	‘It hurts but I can work.’
3. Neutral	‘If I have a headache, can I take ibuprofen?’
4. Positive	‘I was tested and it went well.’
5. Very positive	‘I am very happy, I have not had any discomfort.’
**Themes**	
1. Treatments	‘I will have 12 sessions of chemotherapy lasting 1 hour.’
2. Side effects	‘My hair is going to fall out.’
3. Diagnosis	‘I have HER2 positive breast cancer.
4. Next steps	‘I need a blood test to start treatment.’
5. Doubts	’How can I tell my family?’
6. Family history of cancer	‘Both my grandparents died of cancer.’
7. Medical history	‘I have suffered from atopic dermatitis for 3 years.’
8. Recommendations	‘Use SPF 50 sunscreen all year round.’

The audio recordings of the consultations were transcribed using the Microsoft Word transcription module and revised by one of the research team. Transcriptions were anonymised by replacing all names with the role of the person (ie, patient, oncologist, nurse), and each patient was deidentified with a code referring to the type of cancer and the patient number in the study. The transcriptions analysis and interpretation of the results were carried out using NVivo V.14. The transcripts and the recordings were stored on an external hard drive located in a locked drawer in the office of one of the researchers at the University of Zaragoza.

### Establishing quality in qualitative research

Yardley’s criteria for ensuring the quality of qualitative research were met.^47^ To ensure *sensitivity to context,* previous literature was reviewed before and during the study, and efforts were made to recruit various participant profiles. For c*ommitment and rigour*, each stage of the study and analysis was supervised by other authors to verify that the identified codes, themes and emotions were appropriate. Furthermore, the Standards for Reporting Qualitative Research were followed.^48^ For *transparency and coherence*, the study results are supported by numerous example quotations from the raw data. A reflective diary was used to record representative sentences from each consultation. In addition, the authors articulated their perspectives and approaches regarding the research aim and method. To maximise *impact and importance*, the study results were used to formulate recommendations aimed at enhancing the understanding of patients’ emotional responses during oncological consultations.

## Results

### Participants’ characteristics

Table 2 provides an overview of the participants’ characteristics. Most of the patients were diagnosed with breast cancer and were female, older and from the city of Zaragoza. All of them were starting the oncological process for the first time, except for two patients. Three oncologists participated in the study, predominantly middle-aged men. A total of 12 patients in 31 consultations were recorded with an average duration of 21 min (ranging from 4 to 63). During the recruitment process, 24 patients declined to participate.

**Table 2:** Patient characteristics (n=12)

Characteristics	n (%)
Origin	
City of Zaragoza	10 (83.3)
Rural areas	2 (16.7)
Gender	
Female	9 (75)
Male	3 (25)
Median age (range years)	59 (42–77)
Breast cancer	59
Melanoma	64
Have they been an oncology patient in the past? (>10 years ago)	
Yes	2 (16.7)
Type of cancer	
Breast	8 (66.7)
Melanoma	4 (33.3)
Number of consultations needed to reach a decision	
1 consultation	1 (8.3)
2 consultations	4 (33.3)
3 consultations	6 (50)
4 consultations	1 (8.3)

### Main themes related to patients’ emotional variations

A total of 204 emotional variation phrases were identified across the 31 consultations. Of these, 91 were classified as neutral variations, 75 as negative and 38 as positive. The main themes related to positive or negative emotional variations were medical history, treatment, side effects, diagnostic and next steps. All of them were mentioned by at least half of the patients. Table 3 shows the distribution of emotional variations for each theme. Neutral variations were not considered for the analysis of this study, as they did not report relevant information. Although no significant differences were found between the groups, there were marked negative ratings when discussing diagnosis and medical history. The other three main themes are more balanced, with side effects being the only one that is rated slightly positively.

**Table 3:** Themes and emotional variations matrix

General	Very negative	Negative	Neutral	Positive	Very positive	Total
Medical history	1	24	26	10	0	61
Diagnostics	1	16	12	1	0	30
Treatment	3	10	21	7	1	42
Side effects	0	7	8	10	1	26
Next steps	1	12	24	8	0	45
Total	6	69	91	36	2	204

Themes are sorted in order of appearance in consultations.

#### Medical history

Nine patients, who reported issues with their medical history, were elderly and retired people with previous pathologies. Most of them reported difficulties remembering all the illnesses and their inability to do everything they used to be able to do.

I have so many things that I can’t remember.

Since I retired I've had all kinds of problems … Before, between the shop and the studio I didn't have time for this … boh.

In some cases, the treatment could aggravate their previous pathologies. In all cases, patients prioritised cancer treatment over any other problem. In the example, a patient with a foot disease made it clear that curing his cancer was the most important thing.

I prefer this. We’ll see how it affects the foot. I’ll take whatever anything or do whatever it takes.

#### Diagnostics

This theme appears only in the first oncological consultation. Patients often report their fears and insecurities when they start feeling their tumours as well as the process they went through until their first oncology consultation. Of the seven patients who expressed this, six reported experiencing pain. All cases were preceded by questions from the oncologist regarding information previously provided. Once a question was answered, the subsequent theme was addressed without significantly emphasising the patient’s response. In the following example, the oncologist asks the patient to report what she knew about the diagnosis, and she expresses pain and doubts.

Well, I had breast cancer. I don’t know exactly … but the glands are swollen, and I have orange-peel skin … Then, of course, my leg hurts because the pain in my spine must have passed to my leg, it’s like a tingling, a continuous tingling and pain. I touch it and it feels like it’s asleep.

In other cases related to melanoma, patients often express fear when they start noticing their skin cancer. They talk about the first time they noticed it and the consultation they had with dermatologists.

…I got a little blood, but I didn’t give it any importance because I don’t know, I'm a bit cross and I got blood. But of course, seeing the wart in no time at all, in a short period of time. Yes, I was a little bit worried.

I said, well, if it gets bigger again we’re going to mess it up. I told my dermatologist I was afraid that it would grow like this (melanoma)…

#### Treatment

Seven patients reacted emotionally during the treatment discussion. The most common emotional responses occurred when the recommended course of action differed from previous advice given by other medical professionals. In the following verbatims, strong negative variations and a certain degree of excitement or nervousness may be observed.

But we have suddenly changed the treatment!

Why am I treated with chemotherapy and my daughter is not?

First I was told I was going to have a surgery, but now there is no surgery … but the other times I've had surgery!

Another cause of negativity is when treatment cannot start immediately. For instance, one patient reacted very negatively when informed that their intravenous treatment would be delayed because of the requirement for a peripherally inserted central catheter.

Won’t I get treatment on that Monday? As far as I’m concerned, the sooner I start the better, because it’s taking too long … We are not making any progress, that is, we are not making any progress at all.

The positive emotional variations found are not consistent between patients. Therefore, no clear conclusions can be drawn from them. Positive variations refer to organising treatment to avoid coinciding with holidays, choosing the treatment room and feeling grateful towards the oncologist.

#### Side effects

Patients’ emotional variations to explanations of side effects were predominantly neutral or positive. The latter referred to general side effects of the treatment or the cancer itself. Patients seemed positive and predisposed to go through the process. In most cases, patients were accompanied by their relatives, who may have influenced patients’ positive attitude to avoid causing distress. For example, when an oncologist explains the possible side effects of chemotherapy, patients tend to respond positively, saying that they do not expect to experience any of these effects.

It’s going to be good, you’ll see.

When discussing the possibility of hair loss, patients with breast cancer expressed fear and negativity towards it and towards having to buy a wig. Indeed, many of them asked about the likelihood of this side effect. In contrast to the previous themes, oncologists took time to empathise with patients’ emotions and doubts, responding to all of them positively and clearly. One patient expressed the need to wear a wig to avoid people looking at her and making her feel stigmatised.

It’s not that I wasn’t a supporter, I never wore a wig, not for anything and I always said I wasn't going to wear a wig. But everyone is now telling me that I should think about it. So that people don't look at me.

#### Next steps

Even if the positive and negative variations relating to this theme are balanced, there is a clear difference between them. Patients reported negative emotional variations when a new investigation was required, or when they did not understand the next steps due to the complexity or unclear information. In the quote, the oncologist asked the patient if she knew how to get to the appointment desk and if she would need help. The patient clearly responded that she needed help.

Yes please, because then I’ll make a mess.

Patients were also greatly concerned about the delay in starting treatment due to the need for additional investigations. In the following example, a patient expressed their fear of not starting treatment as soon as possible.

Yes, of course, but it’s going to take a long time … I’m afraid that if I don’t get cured quickly, I'm afraid that it’s going to move on to another gland and…

Additionally, the pain caused by the biopsy and the implantation of biomarkers in the tumours also generated negative variations. As can be seen in the next verbatim, test results generate positive or negative variations. The oncologists did not spend much time explaining the results as patients already knew them.

(echocardiogram test results) ‘So all in order, my heart is fine!’

I was called in for a biopsy. And the result was … Well, it was…

### Visualisation of results

**Figure 1:**
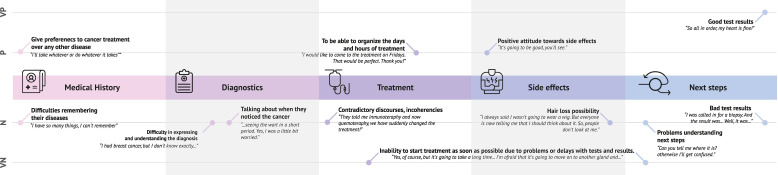
Identified main causes of emotional reactions during the consultation process. n, negative; p, positive; VN, very negative; VP, very positive.

As discussed in this section, the analysed oncology consultations followed a consistent discourse pattern with some minimal variations. Oncologists presented the information in a structured way, following a protocol. They have a similar order to discuss the different themes. This structured approach enabled the clear identification of the emotional variations during the consultations, which can be graphically represented. As can be seen in figure 1, the main causes of emotional variations detected fall into treatment, side effects and next steps. These are all located in the second half of the consultation.

## Discussion

This study examined the experiences and emotional responses of 12 oncology patients during 31 consultations with oncologists, in which the patients were given their diagnoses and treatment options. Two-thirds of the variations were negative. This is not surprising given that cancer is the second leading cause of death.^1^ All of the analysed patients reported any kind of emotional variation. The findings illustrate how patients react emotionally to the main themes discussed in these oncology consultations. Five critical themes emerged, generating mainly negative, but also positive, emotional variations. Patients often expressed fear and distress with issues related to time delays, additional investigations and diagnosis. All the patients expressed their readiness to start the treatment as soon as possible. Any delay was a cause of concern. Complexities related to the next steps that patients need to fulfil generate negative emotions and stress. Conversely, patients remained positive when oncologists explained the side effects. As with other studies, perhaps the presence of relatives influences their attitude as a protective buffering factor.^49^

The study findings are consistent with other previous studies that examine patients’ negative emotions in oncology.^50 51^ These studies examined subsequent stages of cancer care pathways and have reached comparable conclusions. In the study by Andersson *et al*,^50^ they analysed 415 consultations with patients diagnosed with advanced cancer. The consultations were examined to gain insight into how patients expressed their emotions and the themes they discussed. The study identified the following areas as the most prevalent sources of negative emotions among patients: diagnosis, treatment, the effects of treatment and the healthcare system. While these findings align with the themes identified in our study, treatment side effects report more positive than negative variations. This may be because the consultations analysed in this study were conducted before treatment, before patients had experienced any side effects. Unlike other studies, our study provides more insight into patients’ condition at the outset of the oncology process.

Patients with breast cancer in our study reported profoundly negative emotional variations when oncologists explained the possibility of hair loss due to chemotherapy. Our findings are in line with previous literature. For instance, a literature review performed by Boland *et al*^51^ highlights the physical, psychological and social consequences of alopecia in female patients with cancer. For many of these female patients, this side effect is often reported as one of the most traumatic issues of the treatment process, even if it is not the most life-threatening. They usually have problems adapting to their new physical appearance, which might impact their social interactions. While there could be other side effects eliciting strong negative emotional variations, concerns about hair loss were particularly prominent in the initial consultations analysed in our study.

We have not identified any studies that assess the emotions of patients with cancer when discussing their medical history. This may be because most studies focus on later stages of the disease, while these conversations happen primarily at the outset of the cancer process. Another possibility is that medical history is crucial for selecting cancer treatments, but not for treating other illnesses.

This is the first study to analyse patients’ expressed emotional variations in their treatment decision oncology consultations. It provides valuable insight into the experiences and emotions patients face when confronted with the uncertainty of initiating a new oncological process. The methodology and process followed for the analysis of the consultations transcription were similar to other studies and The Verona coding definitions of emotional sequences (VR-CoDES).^37 52^ Nevertheless, some limitations should be considered. The single-centre recruitment model, while allowing for in-depth analysis of one particular process, may restrict the transferability of findings. Although a standardised hospital protocol was followed for communicating key information (such as diagnosis and treatment plans), inherent interpersonal variations in clinician communication style were not controlled for as a variable in the analysis.

Another limitation is the study’s relatively small sample size, which was composed predominantly of female patients. However, data saturation is considered to have been achieved for this specific context. It is supported by the Information Power model^45^ which states that sample adequacy depends on data richness rather than participant numbers alone. Therefore, while generalisability of the results is limited due to the homogeneity of the sample, the robustness and richness of the data collected in this specific context is consistent. However, caution should be exercised when considering the applicability of the results to male patients or more heterogeneous populations.

The identification of emotional variations is a complex process that generates a large amount of relevant data and insights into patients’ experiences.^53^ Although there are various methods of analysing emotions, dimensional analysis was chosen to facilitate consensus and data analysis.^54^ According to the Kappa statistic, consensus among the researchers is quite high. However, this only indicates that the consensus among the researchers is not on the reality of the reaction. It would be necessary to compare the results found with observational notes and interviews with patients to get a more reliable view of emotional variations.

In future research, more patients from other types of cancers should be analysed to have strong conclusions about their emotional variations. Transcription of consultations denies the possibility of capturing the arousal dimension and other emotional expressions such as irony and sarcasm. Analysing the audio of consultations or observing patients’ body language could also provide more insight into their experience and emotions.

### Practical implications

This study reports the themes on which patients react most emotionally during their first oncology consultations. Oncologists should pay more attention to the emotional variations of their patients. This is especially important for themes that are not critical, such as the medical history or additional investigations. Oncological processes are psychologically very demanding; hence, patients should be cared for not only for their illness and its physical effects but also for the psychological effects. Despite the time constraints of consultations, oncologists should approach treatment decisions with greater empathy and understanding. In this regard, figure 1 can serve as a concise reminder of the factors that may lead to emotional fluctuations in patients during consultations.

## Data Availability

Data are available upon reasonable request.
