# Supplementary material for: Identifying emotional variations in Spanish oncology patients during treatment decision consultations: a qualitative study
Source: BMJ Open. 2026 Feb 2;16(2):e099918. doi: 10.1136/bmjopen-2025-099918 (PMC12878401; doi:10.1136/bmjopen-2025-099918)
Supplement: Supplementary file 1 [file bmjopen-2025-099918.reviewer_comments.pdf]

PEER REVIEW HISTORY

BMJ Open publishes all reviews undertaken for accepted manuscripts. Reviewers are asked to complete a checklist review form and are provided with free text boxes to elaborate on their assessment. These free text comments are reproduced below.

ARTICLE DETAILS

Title (Provisional)

Identifying Emotional Variations in Spanish Oncology Patients During Treatment Decision Consultations: A qualitative study

Authors

Sañudo, Yeray; Aguado, Laura; Medina Castillo, Alba; Sierra-Pérez, Jorge

---

VERSION 1 - REVIEW

**Reviewer**	**1**
**Name**	**Taylor, Sally**
**Affiliation**	**The Christie NHS Foundation Trust, Christie Patient Centred Research**
**Date**	**24-Apr-2025**
**COI**	**None**

Thank you for the opportunity to review this interesting paper. Generally, the paper is well written but there are some areas where improvements are needed.

Methods

The authors should provide some information as to why breast and melanoma chosen and a wider number of different disease groups.

The section where the authors discuss recruiting for two reasons does not make sense. Participants have to be recruited to take part in a research study.

The sample size section could be reworded to improve clarity. The first sentence can probably be removed.

It is not clear which consultations were recorded. The authors state in the abstract that participants were followed until they made a treatment decision but this information does not seem to be clear in the main text. Were all new patients approached and then their first consultation recorded. Were subsequent consultations recorded over a set period of time? How did the authors decide how many consultations to record per person?

How many patients were approached to take part and how many declined? If there were decliners, what was their reason for decline?

Results

Can the authors include details of the number of encounters per patient i.e. 5 patients 1 encounter, four patients two encounters etc

Was any ethnicity data collected?

Is the age given a median age? This should be specified

'Expressed doubts about why it did not pain' needs to be reworded as it is currently unclear what this means.

Discussion

Should it say cancer care pathway instead of cancer care path?

Stating that hair loss is the most traumatic side effect for breast cancer patients seems to be a broad generalisation. This is very subjective and may not be the case for all patients. Are the authors just referring to the participants of this study? At present it reads as though this is the most traumatic side effect for all breast cancer patients.

The relatively small sample size of predominantly female patients should be mentioned as a limitation. It seems unlikely that true data saturation was reached with such a small sample that lacked diversity.

**Reviewer**	**2**
**Name**	**Khan, Abdul Rauf**
**Affiliation**	**Gazi University, Department of Mathematics**
**Date**	**30-Jun-2025**
**COI**	**None**

---

1. Abstarct need improvement like don’t start sentence by a number. If some section is not applicable don’t mention it. In some heading used : and in some not use. Remove “:” from all headings in abstract.

2. Introduction section Page 8, Line 11 use (table 1) as (Table 1)

3. Introduction must be improved by citing recent relevant literature

4. At Page 8 Line 15, remove (1=very negative 5=very positive) and refer Table 1.

5. Remove all typo and grammatical errors.

6. Manuscript need improvement overall.

**Reviewer**	**3**
**Name**	**Khayeka−Wandabwa, Christopher**
**Affiliation**	**African Population and Health Research Center (APHRC), Nairobi, Kenya**
**Date**	**15-Oct-2025**
**COI**	**None**

Identifying Emotional Variations in Oncology Patients During Treatment Decision Encounters: A qualitative study

Since there is limited understanding of patients' emotional variations during their first oncology encounters to decide their treatment, this qualitative study aims to explore patients' emotional variations throughout their first oncological encounters to inform which topics discussed create negative and positive emotional variations. It provides insights to improve Patient Center Care (PCC) and discussions between patients and clinicians. More importantly, an analysis of the actual discourse during the consultations could provide further insights into how patients respond, and how they cope with the themes discussed. This is an interesting study in the context of increasing burden of cancers as well as better treatment options promoting longevity of life.

-Page 5 line 58-60 "Eligible participants were required to be new patients in the care paths or have used them more than 10 years ago because they do not know the service process and have no prior expectations." Which care path? Cancer care path or the facility care path or health care path?

-Under methods, were all the patients reviewed by the same or different depending on oncologist(s) in work schedule? If different depending on oncologist on schedule how is inherent interpersonal variation in terns of the oncologist controlled for in the potential outcomes of negative and positive emotional variations?

-"Data processing and analysis" with regard to emotional variations, were the phrase context/text/theme used by the 3 the establish the score the same?

- Please check on few grammar errors e.g. page 9 line 29-31…etc

Overall, the study is well written with clear methodology. It can proceed with minor revisions. It is a good read.

VERSION 1 - AUTHOR RESPONSE

Reviewer: 1

Dr. Sally Taylor, The Christie NHS Foundation Trust
Comments to the Author:
Thank you for the opportunity to review this interesting paper. Generally, the paper is well written but there are some areas where improvements are needed.
Thank you for your interest in our study. We have addressed all the comments that you and other reviewers have raised. Furthermore, the paper has undergone a thorough grammatical review to ensure the removal of any typos.

Methods

The authors should provide some information as to why breast and melanoma chosen and a wider number of different disease groups.

We appreciate your comment and have added an explanation to the article to clarify why we focus on breast cancer and melanoma. This article is related to a European project (4D PICTURE) that aims to redesign care pathways for cancer processes. The pilot studies are focusing on breast cancer, prostate cancer, and melanoma. More specifically, at the Spanish hospital, we are analysing breast cancer and melanoma.

Please see page 4, second paragraph of case study (methods section).

The section where the authors discuss recruiting for two reasons does not make sense. Participants have to be recruited to take part in a research study.

Thank you for your comments. We have rephrased the whole paragraph deleting these reasons and clarifying the recruitment process.

Please see page 5, third paragraph of participants recruitment (method section)

The sample size section could be reworded to improve clarity. The first sentence can probably be removed.

Thank you for your comments and suggestions. The sample size section has been rephrased to improve clarity. We hope it is now easier to understand.

Please see page 5, participants recruitment (method section), lines 14-19

It is not clear which consultations were recorded. The authors state in the abstract that participants were followed until they made a treatment decision but this information does not seem to be clear in the main text. Were all new patients approached and then their first consultation recorded. Were subsequent consultations recorded over a set period of time? How did the authors decide how many consultations to record per person?
We appreciate your comments and have explained more deeply how many consultations were recorded per patient. At no point was it decided how many consultations per patient needed to be recorded. The total number depended on the consultations that oncologists and patients needed to make treatment decisions.

Please see page 5, data collection (method section) lines 21-26

How many patients were approached to take part and how many declined? If there were decliners, what was their reason for decline?
Thank you very much for your question. We have added the total number of patients who declined to participate. In accordance with our committee's ethical guidelines, they were not asked at any time why they did not want to participate. This was considered to be personal information, not relevant to the study.

Please see page 8, participants' characteristics (Results section) line 8

Results

Can the authors include details of the number of encounters per patient i.e. 5 patients 1 encounter, four patients two encounters etc

Following reviewer comments, we have added to Table 2 the number of consultations needed per patient to reach a treatment decision. As can be seen, most of the patients (10) needed between two and three consultations to reach a decision.
Please see page 8, Table 2, participants' characteristics (Results section)

Was any ethnicity data collected?
Dear reviewer. Unfortunately, no data was collected on the ethnicity of the participants, as it was not considered relevant for the study.

Is the age given a median age? This should be specified

We apologise for the misunderstanding. We are indeed referring to the median age of the participants. We have made this clearer in the table, and hope this avoids any further confusion.
Please see page 8, Table 2, participants' characteristics (Results section)
'Expressed doubts about why it did not pain' needs to be reworded as it is currently unclear what this means.
Thank you very much for your comment. To avoid confusion, we have reworded the paragraph and removed the mentioned sentence. As it is not a significant point that has been mentioned in other sections or in the discussion of the study.

Please see page 9, diagnostics (results section) lines 6-7

Discussion

Should it say cancer care pathway instead of cancer care path?

Following the reviewer's suggestion, “cancer care path” has been changed to “cancer care pathway” in the whole document.

Stating that hair loss is the most traumatic side effect for breast cancer patients seems to be a broad generalisation. This is very subjective and may not be the case for all patients. Are the authors just referring to the participants of this study? At present it reads as though this is the most traumatic side effect for all breast cancer patients.

We greatly appreciate the reviewer's comment. It rightly points out that our previous wording could lead to an over-generalization. We have rewritten the paragraph to explain this statement better, making our findings different from other research. We believe the updated version better

reflects the impact of this side effect without making big claims. We hope the text is now clearer and more precise.

Please see page 13, Discussion section lines 30-38

The relatively small sample size of predominantly female patients should be mentioned as a limitation. It seems unlikely that true data saturation was reached with such a small sample that lacked diversity.
Thank you for your comment and appreciation. In line with the reviewer's suggestion, we have revised the paragraph to explicitly state that the small sample size and the predominance of female patients are key limitations for the transferability to other contexts or cancer care pathways. However, we believe that data saturation has been reached in the specific context of the study.

Please see page 14, the Discussion section lines 4-17

Reviewer: 2

Dr. Abdul Rauf Khan, Gazi University
Comments to the Author:
1. Abstarct need improvement like don't start sentence by a number. If some section is not applicable don't mention it. In some heading used : and in some not use. Remove ":" from all headings in abstract.

Thank you for your comments on the abstract. All ":" have been removed from the header. The paragraphs of the abstract have been rewritten to improve its clarity in English. We hope it has improved.

Please see page 1, abstract section

2. Introduction section Page 8, Line 11 use (table 1) as (Table 1)

Thank you for your comment. The typo has been fixed.
3. Introduction must be improved by citing recent relevant literature

We appreciate your comments. We have updated the older references, replacing them with more current ones. Below, you can review the references that have been added and those that have been removed.

Added references:

Kenney, E., & Adhikari, K. (2016). Recent developments in identifying and quantifying emotions during food consumption. Journal of the Science of Food and Agriculture, 96(11), 3627–3630. https://doi.org/10.1002/JSFA.7717

Kirby, E. R., Kenny, K. E., Broom, A. F., Oliffe, J. L., Lewis, S., Wyld, D. K., Yates, P. M., Parker, R. B., & Lwin, Z. (2020). Responses to a cancer diagnosis: a qualitative patient-centred interview study. Supportive Care in Cancer, 28(1), 229–238. https://doi.org/10.1007/s00520-019-04796-z

Tsianakas, V., Maben, J., Wiseman, T., Robert, G., Richardson, A., Madden, P., Griffin, M., & Davies, E. A. (2012). Using patients experiences to identify priorities for quality improvement in

breast cancer care: Patient narratives, surveys or both? BMC Health Services Research, 12(1), 1–11. https://doi.org/10.1186/1472-6963-12-271/TABLES/3

Romeiro, F. B., Pais, V. G., Humphris, G., & Figueiredo-Braga, M. (2025). Patients' emotional expressions and clinicians' responses in oncology – From recognition to exploration of concerns. PEC Innovation, 6, 100374. https://doi.org/10.1016/J.PECINN.2025.100374

Cincidda, C., Pizzoli, S. F. M., Ongaro, G., Oliveri, S., & Pravettoni, G. (2023). Caregiving and Shared Decision Making in Breast and Prostate Cancer Patients: A Systematic Review. Current Oncology, 30(1), 803–823. https://doi.org/10.3390/curroncol30010061

Deleted references:

Bradley, M. M., & Lang, P. J. (1994). Measuring emotion: The self-assessment manikin and the semantic differential. Journal of Behavior Therapy and Experimental Psychiatry, 25(1), 49–59. https://doi.org/10.1016/0005-7916(94)90063-9

Fallowfield, L., Ford, S., & Lewis, S. (1995). No news is not good news: Information preferences of patients with cancer. Psycho-Oncology, 4(3), 197–202. https://doi.org/10.1002/pon.2960040305

Smithson, J. (2000). Using and analysing focus groups: Limitations and possibilities. International Journal of Social Research Methodology, 3(2), 103–119. https://doi.org/10.1080/136455700405172

Fogarty, L. A., Curbow, B. A., Wingard, J. R., McDonnell, K., & Somerfield, M. R. (1999). Can 40 Seconds of Compassion Reduce Patient Anxiety? Journal of Clinical Oncology, 17(1), 371–371. https://doi.org/10.1200/JCO.1999.17.1.371

Pollak, K. I., Arnold, R. M., Jeffreys, A. S., Alexander, S. C., Olsen, M. K., Abernethy, A. P., Sugg Skinner, C., Rodriguez, K. L., & Tulsky, J. A. (2007). Oncologist Communication About Emotion During Visits With Patients With Advanced Cancer. Journal of Clinical Oncology, 25(36), 5748–5752. https://doi.org/10.1200/JCO.2007.12.4180

4. At Page 8 Line 15, remove (1=very negative 5=very positive) and refer Table 1.

Thank you for your comment. It has changed following your suggestion.

5. Remove all typo and grammatical errors.

Thank you for your comment. The paper has undergone a thorough grammatical review to ensure the removal of any typos. We hope it has been improved in clarity and English.

6. Manuscript need improvement overall.

Following this advice, and in careful consideration of all the specific points raised by the reviewers, we have undertaken a comprehensive revision of the document. We believe these collective changes have significantly strengthened the paper. The key improvements include:

1. **Updated and relevant literature:** The introduction has been strengthened by incorporating more recent and relevant references, ensuring the study is framed within the current state of the literature.

Please see the introduction section, pages 2-4.

2. **Enhanced clarity and detail throughout the manuscript**: We have refined the explanations in the Methods section to clarify the recruitment process, sample size rationale, and data collection procedures. Additionally, the entire manuscript has undergone a thorough grammatical review to improve readability.

3. **A more robust and transparent Limitations section:** The study's limitations are now discussed in greater detail. We have provided a clear justification for achieving data saturation within our specific cohort by grounding our rationale in the 'information power' model, while also being more explicit about the limits to the transferability of our findings.

Please see page 14, the Discussion section lines 4-17

4. **Acknowledgement of additional methodological variables:** We have now explicitly acknowledged the potential influence of interpersonal variations among the participating oncologists as a study limitation, adding another layer of critical self-assessment to our methodology.

Please see page 5, data collection (method section) lines 21-26

Please see page 14, the Discussion section lines 4-17

We are confident that these revisions have substantially enhanced the manuscript's clarity, scientific rigor, and overall quality. We appreciate the reviewer's guidance in helping us strengthen our work.

Reviewer: 3

Dr. Christopher Khayeka–Wandabwa, African Population and Health Research Center (APHRC), Nairobi, Kenya
Comments to the Author:
Since there is limited understanding of patients' emotional variations during their first oncology encounters to decide their treatment, this qualitative study aims to explore patients' emotional variations throughout their first oncological encounters to inform which topics discussed create negative and positive emotional variations. It provides insights to improve Patient Center Care (PCC) and discussions between patients and clinicians. More importantly, an analysis of the actual discourse during the consultations could provide further insights into how patients respond, and how they cope with the themes discussed. This is an interesting study in the context of increasing burden of cancers as well as better treatment options promoting longevity of life.

We sincerely appreciate your comment and interest in our study. We greatly value your positive and constructive feedback, which will allow us to improve the study. We trust that we have satisfactorily addressed any concerns or comments you may have had.

-Page 5 line 58-60 "Eligible participants were required to be new patients in the care paths or have used them more than 10 years ago because they do not know the service process and have no prior expectations." Which care path? Cancer care path or the facility care path or health care path?

Thank you for your comment. We have added more information to avoid misunderstandings. We referred to the breast and melanoma cancer care pathways as they are the object of the study.

Please see page 5, participants recruitment (method section) lines 40-41

-Under methods, were all the patients reviewed by the same or different depending on oncologist(s) in work schedule? If different depending on oncologist on schedule how is inherent interpersonal variation in terns of the oncologist controlled for in the potential outcomes of negative and positive emotional variations?

Thank you very much for your feedback. Three oncologists participated in the study. Therefore, the way they communicated with patients may have affected them. However, it is important to note that each patient was always treated by the same oncologist. In addition, the same protocol was followed to communicate key information and discuss the same topics. Therefore, we have added a clarification to both the methodology and the limitations of the study regarding this aspect.

Please see page 5, data collection (method section) lines 21-26

Please see page 5, data collection (method section) lines 21-26

-"Data processing and analysis" with regard to emotional variations, were the phrase context/text/theme used by the 3 the establish the score the same?

Thank you very much for your question. We have rewritten the sentence to make it clearer. Both evaluators analysed the same texts separately, and only the sentences in which both researchers had found emotional variations were taken into account.

Please see page 6, data processing and analysis (method section) lines 6-9

- Please check on few grammar errors e.g. page 9 line 29-31...etc

Thank you for your comment. The paper has undergone a thorough grammatical review to ensure the removal of any typos. We hope it has been improved in clarity and English.

Overall, the study is well written with clear methodology. It can proceed with minor revisions. It is a good read.

VERSION 2 - REVIEW

**Reviewer**	**3**
**Name**	**Khayeka–Wandabwa, Christopher**
**Affiliation**	**African Population and Health Research Center (APHRC), Nairobi, Kenya**
**Date**	**13-Nov-2025**
**COI**	

The authors have handled the revision comments satisfactorily. I hereby recommend accept.
